# Deep learning framework for RNA 5hmC prediction using RNA language model embeddings

**DOI:** 10.1371/journal.pone.0341649

**Published:** 2026-02-03

**Authors:** Md Muhaiminul Islam Nafi

**Affiliations:** 1 Department of CSE, BUET, Dhaka, Bangladesh; 2 Department of CSE, United International University (UIU), Dhaka, Bangladesh; Universita degli Studi della Campania Luigi Vanvitelli, ITALY

## Abstract

By influencing gene expression and contributing to epigenetic modifications, Ribonucleic Acid (RNA) 5-Hydroxymethylcytosine (5hmC) modification significantly affects cellular pathways. It plays an important role in complex regulatory networks and gene expression. Moreover, 5hmC modifications are linked to a variety of human diseases, including diabetes, cancer, and cardiovascular conditions. However, experimental methods to identify RNA 5hmC modifications, such as chromatography and Polymerase Chain Reaction (PCR) amplification, are costly and time-consuming. So, computational methods are necessary to predict these modifications. In this study, several feature descriptors were analyzed and compared to finalize the best ones. Different deep-learning models were explored to design the proposed model architecture. Neighbourhood analysis was conducted on the dataset to provide insights into a deeper understanding of RNA 5hmC modifications. The proposed model, InTrans-RNA5hmC, is a dual-branch deep learning model that has two branches: the Inception branch and the Transformer branch. Word embeddings having the contextual information and language model embeddings from the RiboNucleic Acid Language Model (RiNALMo) were used as the finalized feature descriptors. InTrans-RNA5hmC outperformed existing SOTA methods, achieving 0.97 sensitivity, 0.985 balanced accuracy, and 0.985 F1 score on the Independent test set.

## 1 Introduction

Ribonucleic Acid (RNA) is a single-stranded molecule that plays a vital role in producing cellular proteins and transferring genetic information. RNA is found in all living beings. It comprises a complex collection of molecules that hold and transmit genetic instructions. These instructions are essential for the maintenance of organisms and growth [1]. RNA also facilitates the transfer of genetic information in certain viruses [2]. 100 types of RNA modifications have been detected by several research studies that alter the structure and function of RNA [2–4]. N6-Methyladenosine (m6A) and N7-Methylguanosine (m7G) are some notable examples that regulate different stages of the mRNA lifecycle [5,6]. In the same way, 5-Methylcytosine (5mC) and N1-Methyladenine (m1A) modifications have been found in transfer RNA (tRNA) and ribosomal RNA (rRNA) [7,8]. An important role is played by RNA modifications in regulating cellular processes and functions through actively influencing protein production and gene expression. These modifications reveal the dynamic nature of RNA and provide information about its many biological roles and functions. Of such modifications, 5-Hydroxymethylcytosine (5hmC) is a chemically modified form of cytosine in RNA that is constructed via TET enzyme-mediated oxidation [9]. It is a key transformation that involves cytosine (C) hydroxymethylation. Hydroxymethylation is a chemical reaction that involves the addition of a hydroxymethyl (-CH_2_OH) group to a molecule. For nucleic acids (DNA and RNA), 5-hydroxymethylcytosine (5hmC) results from the hydroxymethylation of cytosine by adding a hydroxymethyl group (-CH_2_OH) at the fifth carbon position (5) of the cytosine ring. It has a significant impact on cellular pathways by contributing to epigenetic changes and altering gene expression [9,10]. The first discovery of 5hmC was in wheat seeds, which highlighted its broad biological relevance. This relevance disclosed 5hmC’s presence across several species and domains and enhanced the understanding of genetic regulation mechanisms [10]. 5hmC plays necessary roles in RNA splicing, translation, and decay in both human and mouse tissues. These further highlighted its significance in gene expression and complex regulatory networks [11]. Furthermore, several human diseases like cancer, diabetes, and cardiovascular disorders are related to 5hmC modifications. It demonstrates its big impact on the health sector. These relations illustrate the potential of 5hmC modifications as biomarkers and targets for medical research and therapeutic interventions [12,13].

Advanced biochemical techniques such as chromatography (Liquid Chromatography-Tandem Mass Spectrometry (LC-MS/MS) [14], High-Performance Liquid Chromatography (HPLC) [15] and Thin-Layer Chromatography (TLC) [16]) and Polymerase Chain Reaction (PCR) [17] amplification are needed to identify 5hmC. These techniques provide precise insights into its structure and function [18]. Despite their accuracy, these techniques are expensive and time-consuming. It poses great challenges for large-scale applications. So, computational methods to identify 5hmC modification in RNA sequences are required. Several computational research studies have been proposed to identify RNA 5-Hydroxymethylcytosine modification. Liu et al. [19] designed a machine learning (ML) model, iRNA5hmC, to predict RNA 5-hydroxymethylcytosine modifications. It is a support vector machine (SVM) [20] model that was trained using two sequence-based feature representations: k-mer spectrum and positional nucleotide binary vector. To identify RNA 5hmC modification, Ahmed et al. [21] proposed a Logistic regression [22] model, iRNA5hmC-PS. They extracted a set of sequence-based features called Position-Specific Gapped k-mer (PSG k-mer) to use as feature descriptors. Ali et al. [23] developed a convolutional neural network (CNN) model named iRhm5CNN that detects RNA 5hmC modification. They utilized the one-hot encoded feature extracted from encoding each nucleotide in the RNA sequences. Zhang et al. [24] constructed iR5hmcSC by combining features from three distinct feature extraction techniques: one-hot encoding, pseudo structure status composition, and k-mer. Additionally, it obtained the best feature results as input to the stacking model using logistic regression (LR) and the chi-squared test. Wibowo et al. [25] designed XGB5hmC, an XGBoost [26] based model, that used the composition of k-spaced nucleic acid pairs (CKSNAP), pseudo-K-tuple nucleotide composition (PseKNC), and position-specific trinucleotide propensity single strand (PSTNPss) as features. Khan et al. [27] proposed a deep learning (DL) model, Deep5HMC, for accurate 5hmC identification. It is a simple feed-forward network model that utilizes seven different sequence-based feature descriptors. Uddin et al. [28] built a machine learning model, XGB5hmC, to detect 5hmC modification. It was trained with the gradient boosting algorithm XGBoost and used five different sequence-based feature descriptors.

Although existing studies contributed much to the research related to RNA 5hmC modification identification, there is still room for improvement. Most of the studies relied on the traditional ML models or just designed a simple deep learning model (simple feedforward neural network) for prediction. Furthermore, most studies utilized only sequence-based features. In recent times, embeddings from pre-trained transformer-based protein language models [29,30] have been great in protein attribute prediction tasks. By generating informative features for both individual proteins and their residues, these pre-trained models provide increased predictive power. Recently, RNA language models such as RiboNucleic Acid Language Model (RiNALMo) [31], RNA-FM [32], and Uni-RNA [33] have been introduced to extract and capture underlying information implicitly embedded within the RNA sequences. Motivated by the success of protein language model embeddings in recent research studies [34,35], embeddings from RiNALMo have been utilized as a feature for the proposed model in this study. We used RiNALMo in our study instead of others, as RiNALMo outperformed RNA-FM and Uni-RNA on various downstream tasks as presented in [31]. To the best of the authors’ knowledge, this study is the first work to employ RNA language model embeddings for the prediction of RNA 5hmC modification. Additionally, a complex and novel deep learning model combining inception [36] and transformer [37] architecture is designed in this work.

It is very challenging to predict RNA 5-Hydroxymethylcytosine (5hmC) modifications because both local and long-range sequence dependencies must be captured. The ability of current techniques, such as convolutional neural networks (CNNs), fully connected neural networks (FNNs), and conventional machine learning (ML)-based models, to accurately model RNA sequences is limited. Because ML-based methods depend on manually created features, they might not be able to generalize to other RNA contexts. CNN-based models struggle to model long-range dependencies, but they are good at capturing local sequence patterns. Although FNN-based models can learn nonlinear relationships, they frequently struggle to capture meaningful sequence structures and lack spatial awareness. To address these challenges,

InTrans-RNA5hmC, a dual-branch model was proposed that integrates Inception modules for local feature extraction and Transformer encoders for global contextual modeling. While the Transformer branch successfully models long-range dependencies in RNA sequences, the Inception branch effectively captures multi-scale sequence patterns. In comparison to conventional single-branch architectures, the proposed model offers a more thorough feature representation by combining the two architectures, which improves predictive performance. Recent studies [38] have also achieved good performance in different research fields by combining a Transformer-based model with convolutional neural networks.

In this study, a novel dual-branch deep learning model, InTrans-RNA5hmC, is constructed that consists of two branches: The Inception branch and the Transformer branch. Word embeddings that have the contextual information of the RNA sequence are given as input to the Inception branch, and RiNALMo embeddings are used as input for the Transformer branch. The features from both branches are combined and used for the final output. The proposed model was compared with different deep-learning models on the validation set. RiNALMo embedding was selected by comparing it to different sequential feature descriptors using 10-fold CV on the Training set. InTrans-RNA5hmC has outperformed existing SOTA models on the Independent test set. It proved the generalization of the proposed model and prevented any overfitting concerns. t-distributed Stochastic Neighbor Embedding (t-SNE) [39] and Uniform Manifold Approximation and Projection (UMAP) [40] plots were illustrated to demonstrate InTrans-RNA5hmC’s performance in separating two different classes.

Lastly, neighbourhood analysis was done on both the Training and Independent datasets. It provided a valuable biological context.

The key contributions of the study are provided below:

- InTrans-RNA5hmC outperformed existing state-of-the-art (SOTA) methods on the Independent test set.
  - In this study, a novel dual-branch deep learning model consisting of Inception and Transformer branches is introduced.
- To the best of the authors’ knowledge, this is the first work to utilize RNA language model embeddings for RNA 5hmC modification prediction.
- InTrans-RNA5hmC was evaluated via validation and independent datasets to thoroughly investigate model overfitting and its generalization.
  - Neighbourhood analysis was done using Shapley Additive Explanations (SHAP) [41], Two Sample Logo (TSL) [42], nucleotide count, and frequency plots.
- The study explores and discusses some biological implications and hypotheses related to the observed patterns. It is hoped to contribute to a deeper understanding of RNA 5hmC modification.

The following is the arrangement of the remaining sections. The materials and methods utilized in this paper are described in Section 2. The findings of several experiments, performance assessments, comparisons of methodologies, and a neighbourhood analysis are shown in Section 3. The implications of the findings related to RNA 5hmC modification prediction are examined in Section 4. Section 5 wraps up the paper with possible future directions.

## 2 Materials and methods

In this section, datasets, feature extraction, the performance metrics used in this study, the selection processes of the feature descriptor and the final model, and the proposed model architecture are described.

### 2.1 Dataset

The dataset summary is provided in Table 1. In this study, the original dataset from [19] was used. Liu et al. constructed the positive and negative samples based on the hMeRIP-seq [43] method. Each sample had a length of 41 nt (nucleotides) with the 5hmC or non-5hmC site (cytosine (C)) in the center. The experimentally verified original dataset had 662 5hmC sites (positive samples) and 662 non-5hmC sites (negative samples). The original dataset had a pairwise sequence identity of less than 20% to minimize redundancy. In this study, CD-HIT [44] was applied to further check and verify this threshold to ensure a proper evaluation of the proposed model, and the original dataset was split into two sets: Training and Independent test sets. 90% of the original dataset was assigned as the **Training set** and the remaining 10% was assigned as the **Independent test set**. The Independent test set was held out from the beginning, while the Validation set was split only from the Training set. The Training set had 595 positive and 595 negative samples. Whereas the Independent test set consists of 67 positive and 67 negative samples. For the evaluation of different deep learning models in Section 3.2, 80% of the Training set was used to train the model, and the rest 20% was used as the **Validation set** for evaluation.

**Table 1:** Dataset summary.

Dataset	Positive samples	Negative samples	Total samples
Training	595	595	1190
Independent	67	67	134

### 2.2 Feature extraction

In this subsection, the descriptions of feature descriptors and their extraction processes are illustrated. The feature descriptors explored in this study and their feature sizes are provided in Table 2.

**Table 2:** Feature descriptors and their size. Here, the sample is the number of samples.

Feature descriptors	Size
RiNALMo	(sample, 41, 1280)
Word embeddings	(sample, 41, 32)
ANF	(sample, 41)
binary	(sample, 164)
DAC	(sample, 12)
DNC	(sample, 16)
EIIP	(sample, 41)
ENAC	(sample, 148)
Kmer	(sample, 16)
NAC	(sample, 4)
NCP	(sample, 123)
PCPseDNC	(sample, 18)
PseDNC	(sample, 18)
PseEIIP	(sample, 64)
RCKmer	(sample, 10)
TNC	(sample, 64)

#### Per-nucleotide embeddings from RiNALMo (RiNALMo).

With 650M parameters pre-trained on 36M RNA sequences from several databases, the RiboNucleic Acid Language Model (RiNALMo) [31] is the largest RNA language model to date. It is capable of capturing the underlying structural information that is implicitly encoded in RNA sequences and extracting hidden knowledge. The 650M parameter BERT-style Transformer encoder RiNALMo was developed using modern architectural methods like FlashAttention-2 [45], SwiGLU activation function [46], and rotary positional embedding (RoPE) [47]. The feature size of the embeddings is 1280. In this study, per-nucleotide embeddings were generated from RiNALMo for each 41-nt length sample. This (41, 1280) size tensor was taken as input for the Transformer branch of the proposed final model.

#### Word embeddings.

The idea of generating such embeddings was inspired by [34]. To generate the Word embeddings, each nucleotide in the 41-nucleotide (nt) sample was mapped to an integer: ‘A’ (Adenine) to 0, ‘C’ (Cytosine) to 1, ‘U’ (Uracil) to 2, and ‘G’ (Guanine) to 3. For every 41-nt sample, this integer encoding transforms the nucleotide sequence into a fixed-length vector of integers with a size of 41. These integer-encoded sequences were sent to the Inception branch to exploit local patterns among the neighboring nucleotides. An embedding layer transforms the original integer encoding into 32-size embedding vectors for each nucleotide in the 41-nucleotide (nt) sample. Thus, the final feature dimension of the Word embeddings per sample is (41, 32).

The two feature descriptors above were used as input to the final proposed model. However, other feature descriptors were explored in this study. Those are the following: Accumulated nucleotide frequency (ANF) [48], Binary (binary) [48], Dinucleotide-based auto covariance (DAC) [48], Di-nucleotide composition (DNC) [48], Electron-ion interaction pseudopotentials value (EIIP) [48], Enhanced nucleic acid composition (ENAC) [48], Basic kmer (Kmer) [48], Nucleic acid composition (NAC) [48], Nucleotide chemical property (NCP) [48], Parallel correlation pseudo dinucleotide composition (PCPseDNC) [48], Pseudo dinucleotide composition (PseDNC) [48], Electron-ion interaction pseudopotentials of trinucleotide (PseEIIP) [48], Reverse complement kmer (RCKmer) [48] and Tri-nucleotide composition (TNC) [48]. These feature descriptors were extracted using the iLearn [48] tool.

### 2.3 Performance metrics

A well-known set of performance metrics [49–52], including the Matthews Correlation Coefficient (MCC), Sensitivity (SN), Specificity (SP), Accuracy (ACC), Balanced Accuracy (BACC), Precision (PREC), F1 Score (F1), Area Under the Precision-Recall Curve (AUPR), and Area Under the Curve (AUC), were used to assess how well the model predicted the binary labels. Below are the formulas used to determine these scores. True Positives (TP), False Positives (FP), True Negatives (TN), and False Negatives (FN) are the terms used in the formulas.

#### Sensitivity (SN).

Sensitivity (SN) measures the proportion of actual positives that were correctly identified by the model.


$$SN=\frac{TP}{TP\;+\;FN}$$ (1)

#### Specificity (SP).

Specificity (SP) measures the proportion of actual negatives that were correctly identified by the model.


$$SP=\frac{TN}{TN\;+\;FP}$$ (2)

#### Accuracy (ACC).

Accuracy (ACC) is the overall proportion of correctly classified samples (both positive and negative) out of all samples.


$$ACC=\frac{TP\;+\;TN}{FP\;+\;TP\;+\;TN\;+\;FN}$$ (3)

#### Balanced Accuracy (BACC).

Balanced Accuracy (BACC) is the mean of SN and SP.


$$BACC=\frac{SN\;+\;SP\;}{\text{2}}$$ (4)

#### Precision (PREC).

Precision (PREC) measures the proportion of samples that were correctly classified as positive out of all samples classified as positive by the model.


$$PREC=\frac{TP}{FP\;+\;TP}$$ (5)

#### F1 Score (F1).

F1 Score (F1) is the harmonic mean of PREC and SN.


$$\text{F1}=\frac{\text{PREC}\;\times \;\text{SN}\;}{\text{PREC}\;+\;\text{SN}}=\frac{\text{2}TP}{\text{2}TP+FP+FN}$$ (6)

#### Matthews Correlation Coefficient (MCC).

Matthews Correlation Coefficient (MCC) is a measure of the quality of binary classifications. MCC provides a balanced view of performance. It takes both true and false positives and negatives into account.

### 2.4 Model architecture

A dual-branch deep learning model, InTrans-RNA5hmC, consisting of Inception and Transformer branches, was proposed as the final model. The complete model architecture is illustrated in Fig 2.

**Fig 1:**
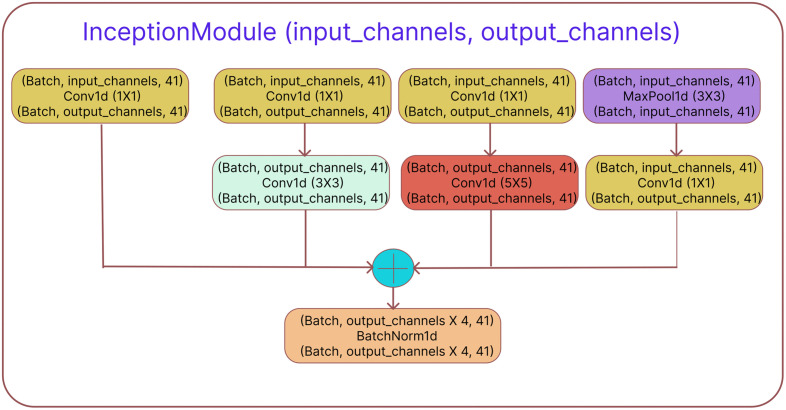
The architecture of the Inception module. It has four parallel convolutional paths. Each path uses different kernel sizes in the Conv1D layers. Lastly, the outputs from the four paths are concatenated along the channel dimension to form the final output. In each three-line block, the first line represents the input tensor size, the second line represents the layer name, and the third line represents the output tensor size. Batch refers to the batch size.

#### Inception branch.

**Fig 2:**
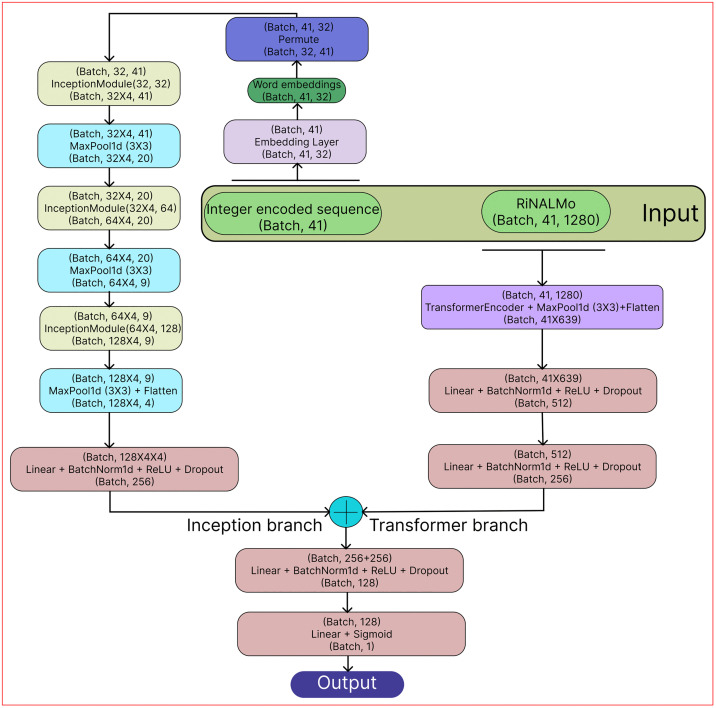
Model architecture of the proposed model, InTrans-RNA5hmC. There are two input embeddings: Word embeddings and RiNALMo embeddings. The model has two branches: The Inception branch and the Transformer branch. The Word embeddings and RiNALMo embeddings are fed to the Inception branch and the Transformer branch, respectively. The features from both branches are concatenated and passed through a feed-forward neural network for final predictions. In each three-line block, the first line represents the input tensor size, the second line represents the layer name and the third line represents the output tensor size. Batch refers to the batch size.

This branch used the Word embeddings as input. The inception module, which is shown in Fig 1, was utilized in this instance. The multi-scale contextual information found in the Word embeddings is intended to be captured by the inception module. In Fig 1, an inception module has four convolutional paths. In path 1, low-level features are captured using a straightforward 1x1 convolution (with kernel size 1). Medium-range features are captured in path 2 by a 1x1 convolution and a 3x3 convolution. In path 3, a 1x1 convolution followed by a 5x5 convolution improves the capacity to capture long-range dependencies, while in path 4, a max pooling operation followed by a 1x1 convolution helps aggregate crucial information. The outputs of these four paths are concatenated along the channel dimension and then subjected to batch normalization to speed up convergence and enhance generalization. To create a 32-size Word embedding vector for each of the 41 nucleotides, the integer encoded sequences are first sent to an embedding layer. The embedding layer generates the Word embeddings. The embedding layer maps each integer (representing a nucleotide) to a continuous 32-dimensional vector. It captures the meaningful relationships between nucleotides in a learned representation. These embeddings serve as input to the Inception branch. This enables the model to extract local patterns among neighboring nucleotides. After that, these Word embeddings go through three inception modules in succession. The model can learn both fine-grained and broader representations of the embedding vectors thanks to the combination of multiple convolutional paths with varying kernel sizes in each of the inception modules. To minimize spatial dimensions, max-pooling layers come after these inception modules. These features are then flattened and fed into a linear layer, which produces a tensor of size (batch size, 256) that can be seen in the Inception branch of Fig 2.

#### Transformer branch.

In this branch, RiNALMo embeddings were used as input. In Fig 2, the RiNALMo embeddings are processed using a series of Transformer encoder layers. The embeddings are passed through a stack of 6 Transformer encoder layers. Each encoder layer utilizes multi-head self-attention and position-wise feedforward networks. It captures long-range dependencies in the RNA sequence. After the attention mechanism, the output undergoes a max-pooling operation to reduce the feature dimension. Then it is flattened and passed through fully connected layers, which produces a tensor of size (batch size, 256) that can be found in the Transformer branch of Fig 2.

The resulting features from both the Inception and Transformer branches are concatenated to integrate both local and global contextual information. From Fig 2, it can be seen that the concatenated features are then fed to two consecutive linear layers. ReLU activation was used to enhance gradient flow in the network, dropout was used to lessen overfitting, and batch normalization was used to stabilize training in each linear layer of the model. The model was trained using the Adam optimizer (Adaptive Moment Estimation). The configuration of the proposed model, InTrans-RNA5hmC, is provided in Table 3.

**Table 3:** Model configuration of InTrans-RNA5hmC.

Name of the parameters	Values
Input shape (Inception branch)	(Batch size, 41)
Input shape (Transformer branch)	(Batch size, 41, 1280)
Output shape	(Batch size, 1)
Activation Function (except output layer)	ReLU
Activation Function (output layer)	Sigmoid
Optimizer	Adam
Epoch	18
Initial learning rate	0.00001
Loss function	Binary Cross-Entropy Loss
Batch normalization	1D
Batch Size	500
Dropout (Inception branch)	0.5
Dropout (except Inception branch)	0.3
Maximum L2 norm	1

**Table 4:** Tuned hyperparameter values from GridSearch conducted on InTrans-RNA5hmC.

Name of the parameters	Search Values	Tuned Values
Epoch	15, 18, 20	18
Dropout (Inception branch)	0.3, 0.4, 0.5	0.5
Dropout (except Inception branch)	0.3, 0.4, 0.5	0.3

### 2.5 Feature descriptor and model selection

For generating the contextual information from the 41-nt RNA samples, Word embeddings were fixed as the input to the Inception branch of the model architecture. The selection of feature descriptors was applied to the other feature descriptors.

Extreme Gradient Boosting (XGBoost or XGB) [26] is proven to be an effective prediction model in diverse fields [53–57]. So, XGB was used for feature selection. An XGB model was trained individually using each of these feature descriptors. 10-fold CV performance results were generated to evaluate the feature descriptors. As RiNALMo generates embeddings with a tensor size of (batch size, seq length, embedding size), the embeddings were averaged along the sequence length dimension to make it suitable for input into the XGB model. In Table 5, the XGB model, trained using RiNALMo embeddings, showed superior performance to other feature descriptors.

**Table 5:** 10-fold CV performance results of different feature descriptors. The scores are presented in ‘mean±standard deviation’ format. An XGB model was trained using each one of the feature descriptors separately on the Training set. The highest values of each metric are boldfaced. As RiNALMo generates embeddings with a tensor size of (batch size, seq length, embedding size), the embeddings were averaged along the sequence length dimension for comparison.

Feature descriptors	SN	SP	ACC	BACC	PREC	F1	MCC	AUC	AUPR
RiNALMo (averaged)	**0.608** ± 0.017	**0.597** ± 0.015	**0.603** ± 0.017	**0.603** ± 0.016	**0.601** ± 0.012	**0.605** ± 0.015	**0.205** ± 0.014	**0.642** ± 0.020	**0.625** ± 0.020
NCP	0.503 ± 0.021	0.548 ± 0.013	0.525 ± 0.019	0.525 ± 0.018	0.526 ± 0.012	0.514 ± 0.017	0.050 ± 0.012	0.512 ± 0.011	0.506 ± 0.017
PCPseDNC	0.580 ± 0.017	0.551 ± 0.019	0.566 ± 0.014	0.566 ± 0.016	0.564 ± 0.017	0.572 ± 0.018	0.131 ± 0.015	0.577 ± 0.018	0.567 ± 0.017
PseDNC	0.580 ± 0.017	0.551 ± 0.013	0.566 ± 0.011	0.566 ± 0.015	0.564 ± 0.015	0.572 ± 0.016	0.131 ± 0.017	0.577 ± 0.016	0.567 ± 0.015
PseEIIP	0.578 ± 0.015	0.587 ± 0.015	0.582 ± 0.019	0.582 ± 0.013	0.583 ± 0.016	0.581 ± 0.014	0.165 ± 0.013	0.625 ± 0.016	0.616 ± 0.020
RCKmer	0.543 ± 0.014	0.561 ± 0.013	0.552 ± 0.015	0.552 ± 0.014	0.553 ± 0.016	0.548 ± 0.016	0.104 ± 0.013	0.566 ± 0.020	0.559 ± 0.016
TNC	0.578 ± 0.017	0.587 ± 0.017	0.582 ± 0.020	0.582 ± 0.015	0.583 ± 0.016	0.581 ± 0.012	0.165 ± 0.012	0.625 ± 0.018	0.616 ± 0.020
ANF	0.499 ± 0.022	0.519 ± 0.018	0.509 ± 0.015	0.509 ± 0.020	0.509 ± 0.014	0.504 ± 0.017	0.018 ± 0.017	0.518 ± 0.019	0.527 ± 0.018
binary	0.513 ± 0.020	0.516 ± 0.021	0.514 ± 0.020	0.514 ± 0.014	0.514 ± 0.015	0.513 ± 0.015	0.029 ± 0.019	0.517 ± 0.017	0.511 ± 0.018
EIIP	0.541 ± 0.017	0.538 ± 0.011	0.539 ± 0.019	0.539 ± 0.016	0.539 ± 0.017	0.540 ± 0.015	0.079 ± 0.019	0.541 ± 0.015	0.528 ± 0.018
ENAC	0.582 ± 0.018	0.531 ± 0.018	0.556 ± 0.020	0.556 ± 0.017	0.554 ± 0.014	0.567 ± 0.020	0.113 ± 0.017	0.586 ± 0.018	0.601 ± 0.016
Kmer	0.578 ± 0.019	0.561 ± 0.017	0.570 ± 0.014	0.570 ± 0.013	0.569 ± 0.017	0.573 ± 0.019	0.140 ± 0.018	0.605 ± 0.021	0.607 ± 0.019
NAC	0.543 ± 0.021	0.529 ± 0.013	0.536 ± 0.013	0.536 ± 0.020	0.536 ± 0.017	0.539 ± 0.013	0.072 ± 0.010	0.527 ± 0.017	0.521 ± 0.014
DAC	0.593 ± 0.018	0.560 ± 0.015	0.576 ± 0.017	0.576 ± 0.017	0.574 ± 0.017	0.583 ± 0.021	0.153 ± 0.019	0.593 ± 0.019	0.568 ± 0.022
DNC	0.578 ± 0.017	0.561 ± 0.023	0.570 ± 0.019	0.570 ± 0.017	0.569 ± 0.021	0.573 ± 0.018	0.140 ± 0.017	0.605 ± 0.016	0.607 ± 0.019

As this study is for predicting RNA 5hmC modifications, the deep learning models effective for sequential data were explored, including Inception, Recurrent Neural Networks (RNNs) [58], Long Short-Term Memory (LSTM) [59], and Transformer architectures. The proposed model architecture has two branches. Pairwise combinations of Inception, RNN, LSTM, and Transformer architectures were utilized in two different branches. These combinations were trained on 80% of the Training set and tested on the Validation set (the remaining 20%). It was repeated 20 times with different random seeds. In Table 6, ‘Inception+Transformer’ outperformed other combinations. Additionally, this model was compared with traditional machine learning (ML) models such as support vector machine (SVM) [60], extreme gradient boosting (XGB), logistic regression (LR) [22], and k-nearest neighbors (KNN) [61]. These experiments were also repeated 20 times with different random seeds. The classification threshold was set to 0.5 for all models used in this study.

**Table 6:** Performance results of different deep learning models which were trained on the 80% Training set and tested on the Validation set (the remaining 20% Training set). The highest values of each metric are boldfaced. The scores are presented in ‘mean±standard deviation’ format. Here, in the {DL1 + DL2} architecture, Word embeddings are used as input to the DL1 branch, and RiNALMo embeddings are used as input to the DL2 branch. This experiment was repeated 20 times with different random seeds.

Model	SN	SP	ACC	BACC	PREC	F1	MCC	AUC	AUPR
Transformer+Transformer	0.855 ± 0.017	0.887 ± 0.015	0.870 ± 0.017	0.871 ± 0.016	0.903 ± 0.012	0.878 ± 0.015	0.740 ± 0.014	0.871 ± 0.020	0.919 ± 0.020
LSTM+Transformer	0.740 ± 0.022	0.804 ± 0.018	0.769 ± 0.015	0.772 ± 0.020	0.822 ± 0.014	0.779 ± 0.017	0.541 ± 0.017	0.772 ± 0.019	0.853 ± 0.018
RNN+Transformer	0.893 ± 0.020	0.822 ± 0.021	0.861 ± 0.020	0.858 ± 0.014	0.860 ± 0.015	0.876 ± 0.015	0.719 ± 0.019	0.858 ± 0.017	0.906 ± 0.018
Inception+Transformer	**0.947** ± 0.018	**0.888** ± 0.015	**0.920** ± 0.017	**0.917** ± 0.017	**0.912** ± 0.017	**0.929** ± 0.021	**0.839** ± 0.019	**0.917** ± 0.019	**0.944** ± 0.022
Inception+Inception	0.901 ± 0.017	0.561 ± 0.023	0.748 ± 0.019	0.731 ± 0.017	0.715 ± 0.021	0.797 ± 0.018	0.498 ± 0.017	0.731 ± 0.016	0.835 ± 0.019
Inception+LSTM	**0.985** ± 0.017	0.570 ± 0.011	0.798 ± 0.019	0.777 ± 0.016	0.737 ± 0.017	0.843 ± 0.015	0.626 ± 0.019	0.777 ± 0.015	0.865 ± 0.018
Inception+RNN	0.863 ± 0.018	0.692 ± 0.018	0.786 ± 0.020	0.777 ± 0.017	0.774 ± 0.014	0.816 ± 0.020	0.566 ± 0.017	0.777 ± 0.018	0.856 ± 0.016

### 2.6 Hyperparameter tuning

Hyperparameter tuning was initially performed through trial and error. The parameter values were selected based on the author’s research experience and performance metrics on the validation set. From this process, the initial learning rate was set to 0.00001, and the batch size was set to 500. A couple of values were explored, and a GridSearch was conducted for the parameters: epoch, dropout (Inception branch), and dropout (outside the Inception branch). The search values and tuned hyperparameters are provided in Table 4. Performance metrics generated from the GridSearch are given in the Supplementary material. The hyperparameters that achieved the best performance scores in F1, MCC, AUC, and AUPR were selected.

## 3 Results

This section depicts the experiments and their results conducted in this study.

### 3.1 Feature selection

As described in Section 2.5, 10-fold CV performance results of different feature descriptors (as listed in Section 2.2) were generated on the Training set. An XGB model was trained using these feature descriptors individually. The results are shown in Table 5. In Table 5, RiNALMo embeddings were averaged along the sequence length dimension to make it suitable for the XGB model input. We can see that the XGB model, trained using RiNALMo embeddings, performed the best in all metrics. Therefore, RiNALMo embeddings were selected as the final feature descriptor of the proposed model. We conducted the pairwise Wilcoxon signed-rank tests between RiNALMo (averaged) and the other remaining feature descriptors to determine the meaningfulness of RiNALMo’s highest performance. All p-values were less than 0.05, with an average p-value of 0.00017, which is less than 0.05. So, the improvements were statistically meaningful.

### 3.2 Validation results

As described in Section 2.5, several deep learning methods were explored to get the best-performing model. Given the proposed model’s dual-branch architecture, pairwise combinations of Inception, RNN, LSTM, and Transformer models were trained on 80% of the training set and evaluated on the Validation (remaining 20%) set. This experiment was repeated 20 times with different random seeds. The performance results are provided in Table 6. Firstly, Transformer+Transformer, LSTM+Transformer, RNN+Transformer, and Inception+Transformer pairwise combinations were analyzed. In the *{*DL1 + DL2*}* architecture, the DL1 branch receives Word embeddings as input, whereas the DL2 branch receives RiNALMo embeddings. From the results, we can see that Inception+Transformer obtained the highest scores in all metrics among the four combinations. So, Inception was finalized for the input branch of Word embeddings.

Subsequently, Inception+Transformer, Inception+Inception, Inception+LSTM, and Inception+RNN pairwise combinations were analyzed. Once again, Inception+Transformer was the top-performing model across nearly all metrics. So, Inception+Transformer was finalized as the proposed model, **InTrans-RNA5hmC**. We also performed pairwise Wilcoxon signed-rank tests between Inception+Transformer and each of the other models. The average p-values were 0.01 in Table 6 and 9.537e-7 in Table 7, both of which are below the 0.05 threshold. It indicates that the performance improvements are statistically significant.

**Table 7:** Performance results of different ML models which were trained on the 80% Training set and tested on the Validation set (rest 20% Training set). The scores are presented in ‘mean±standard deviation’ format. The highest values of each metric are boldfaced. This experiment was repeated 20 times with different random seeds.

Model	SN	SP	ACC	BACC	PREC	F1	MCC	AUC	AUPR
Inception+Transformer	**0.947** ± 0.018	**0.888** ± 0.015	**0.920** ± 0.017	**0.917** ± 0.017	**0.912** ± 0.017	**0.929** ± 0.021	**0.839** ± 0.019	**0.917** ± 0.019	**0.944** ± 0.022
SVM	0.527 ± 0.017	0.607 ± 0.015	0.563 ± 0.017	0.567 ± 0.016	0.622 ± 0.012	0.570 ± 0.015	0.134 ± 0.014	0.574 ± 0.020	0.633 ± 0.020
KNN	0.573 ± 0.020	0.561 ± 0.021	0.567 ± 0.020	0.567 ± 0.014	0.615 ± 0.015	0.593 ± 0.015	0.133 ± 0.019	0.598 ± 0.017	0.645 ± 0.018
Logistic Regression	0.611 ± 0.022	0.579 ± 0.018	0.597 ± 0.015	0.595 ± 0.020	0.640 ± 0.014	0.625 ± 0.017	0.189 ± 0.017	0.603 ± 0.019	0.657 ± 0.018
XGBoost	0.588 ± 0.017	0.607 ± 0.023	0.597 ± 0.019	0.598 ± 0.017	0.647 ± 0.021	0.616 ± 0.018	0.194 ± 0.017	0.657 ± 0.016	0.667 ± 0.019

**Table 8:** Comparison with the SOTA methods on the Independent dataset. The highest values of each metric are boldfaced. Metrics not reported by the respective papers are indicated as’-’ in the table.

SOTA	SN	SP	ACC	BACC	PREC	F1	MCC	AUC	AUPR
iRNA5hmC	0.677	0.633	0.655	0.655	–	0.65	0.31	–	–
iRNA5hmC-PS	0.8	0.795	0.783	0.783	–	0.764	0.56	–	–
iRhm5CNN	0.82	0.8	0.81	0.81	–	0.78	0.62	–	–
iR5hmcSC	0.824	0.884	0.853	0.854	–	–	0.709	–	–
XGB5hmC [25]	**0.982**	0.973	0.977	0.977	–	–	0.955	–	–
Deep5hmC	0.903	0.831	0.841	0.841	–	0.839	0.736	–	–
XGB5hmC [28]	0.878	0.945	0.9	0.9	–	0.893	0.876	–	–
InTrans-RNA5hmC	0.97	**1**	**0.985**	**0.985**	**1**	**0.985**	**0.971**	**0.985**	**0.993**

**Table 9:** Ablation study results: performance comparison of Inception-only, Transformer-only, and InTrans-RNA5hmC models. Models were trained on the Training set and tested on the Independent test set.

Model	SN	SP	ACC	BACC	PREC	F1	MCC	AUC	AUPR
Inception-only	0.955	0.716	0.836	0.836	0.771	0.853	0.692	0.836	0.874
Transformer-only	**0.97**	0.91	0.94	0.94	0.915	0.942	0.882	0.94	0.950
InTrans-RNA5hmC	**0.97**	**1**	**0.985**	**0.985**	**1**	**0.985**	**0.971**	**0.985**	**0.993**

Additionally, as described in Section 2.5, Inception+Transformer was compared with traditional ML models (SVM, XGB, LR, and KNN). The comparison is provided in Table 7. This was repeated 20 times with different random seeds. RiNALMo embeddings were averaged along the sequence length dimension to make it suitable as input for the traditional ML models. In Table 7, Inception+Transformer outperformed other models in all performance metrics. This supports the initial hypothesis stated in Section 2.5 that deep learning models designed for sequential data are more effective for predicting RNA 5hmC modifications.

Furthermore, we evaluated the model, ‘Inception+Transformer’, using 10-fold cross-validation on the whole dataset. The model achieved strong performance across all evaluation metrics, including SN (0.933), SP (1.000), ACC (0.967), BACC (0.967),

PREC (1.000), F1 (0.965), MCC (0.936), AUC (0.967), and AUPR (0.983). To assess the consistency of performance across folds, we conducted Friedman tests for each evaluation metric. In all cases, the resulting p-values were greater than 0.05, indicating no statistically significant differences across folds and confirming the robustness and stability of the model’s performance.

### 3.3 Comparison with existing SOTA methods using the Inde- pendent test set

The proposed model, InTrans-RNA5hmC, was compared with other existing SOTA methods on the Independent test set in Table 8. From the table, we can see that InTrans-RNA5hmC achieved the highest scores in almost all performance metrics. InTrans-RNA5hmC outperformed the existing SOTA methods. An ablation study was conducted to compare the performance of three model variations: the Inception-only, the Transformer-only, and the complete InTrans-RNA5hmC model. The results are provided in Table 9, which further validates the effectiveness of the proposed model, InTrans-RNA5hmC. InTrans-RNA5hmC outperforms individual branches, which highlights the supplementary strengths of both the Inception and Transformer branches. Moreover, the results demonstrate the advantage of integrating both word embeddings and RiNALMo embeddings, as the combined approach surpasses models that rely solely on word embeddings (Inception-only) or solely on RiNALMo embeddings (Transformer-only).

#### 3.4t-SNE and UMAP visualization of the feature space.

**Fig 3:**
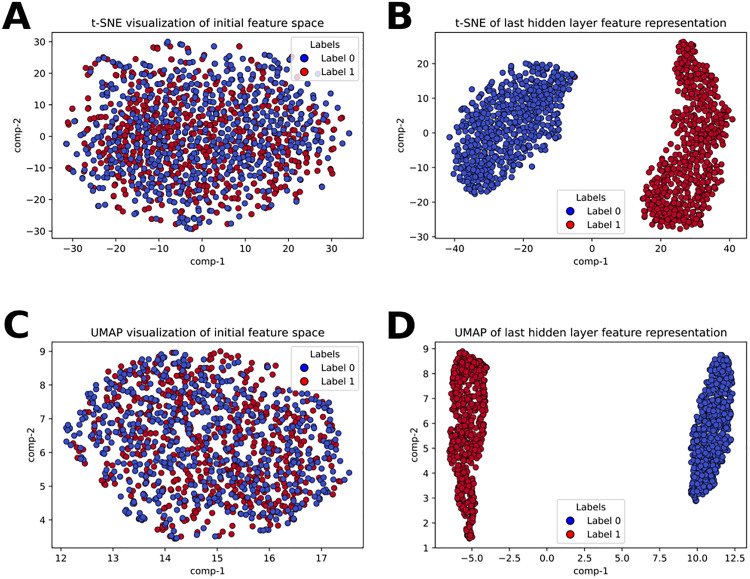
t-SNE and UMAP visualizations of the proposed InTrans-RNA5hmC model on the Training dataset. The first row shows the initial vs. learned feature representation using t-SNE. The second row shows the same result using UMAP. A: t-SNE visualization of the initial feature space, B: t-SNE visualization of the last hidden layer feature representation of the InTrans-RNA5hmC model, C: UMAP visualization of the initial feature space, D: UMAP visualization of the last hidden layer feature representation of the InTrans-RNA5hmC model.

t-SNE and UMAP visualizations on the feature space are illustrated in Fig 3. For both visualizations, the first plot in each row displays the initial feature space of the samples, and the second plot displays the last hidden layer feature representation of the InTrans-RNA5hmC model. From the figures, we can say that InTrans-RNA5hmC reasonably separates the different class samples. It demonstrates the model’s ability to learn meaningful and discriminative representations in the feature space.

### 3.5 Neighbourhood analysis

**Fig 4:**
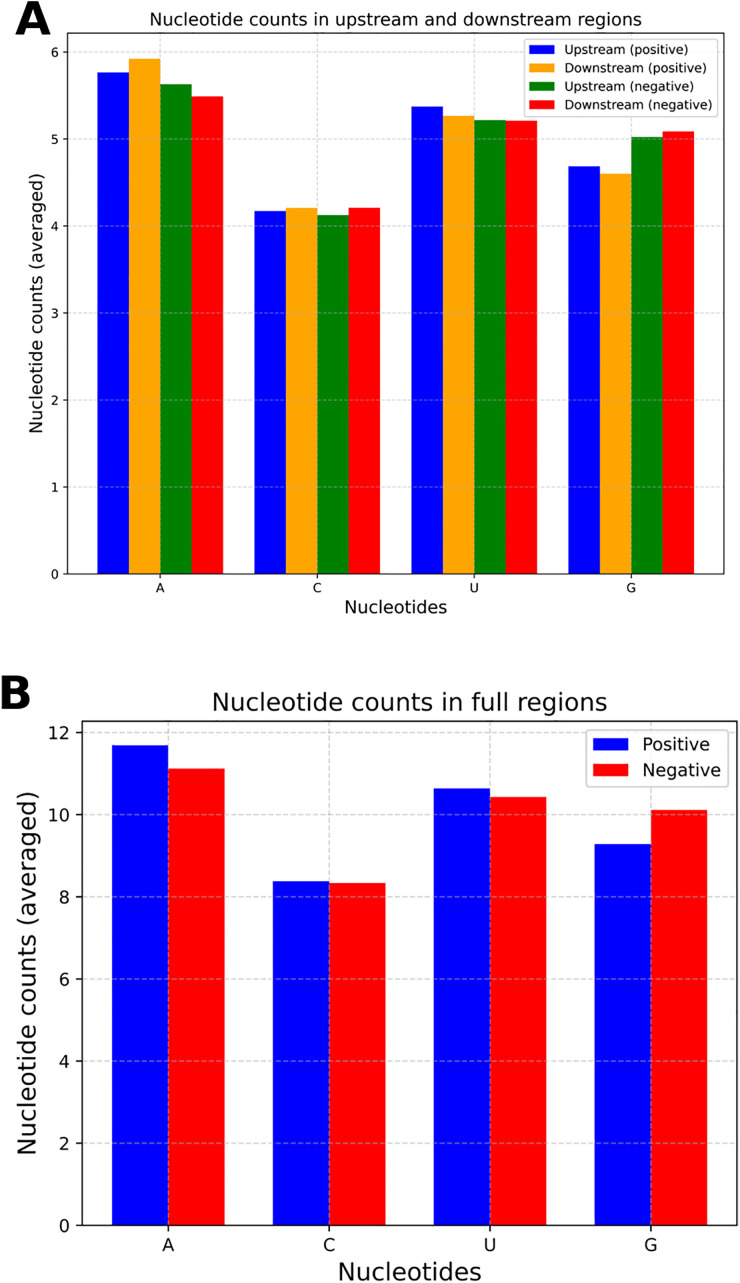
Comparison of nucleotide count distributions across upstream, downstream, and full regions. A: Bar plot of nucleotide counts in upstream and downstream regions. As the sequence length was 41 and the central nucleotide was in position 20, the upstream region spans positions 0 to 19, and the downstream region covers positions 21 to 40. The nucleotide counts were averaged across all sequences. B: Bar plot of nucleotide counts in full regions. The full region refers to the whole sequence. The nucleotide counts were averaged across all sequences.

**Fig 5:**
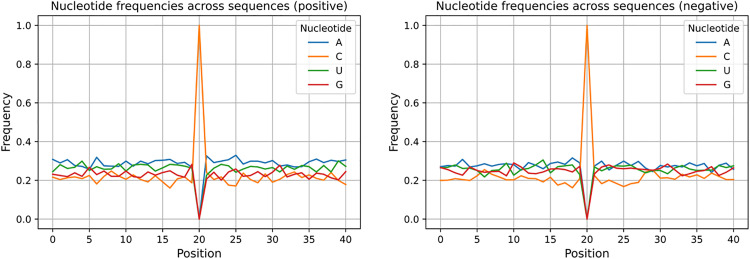
Plots of nucleotide frequencies at each position across sequences for both positive and negative samples. The first plot belongs to the positive samples, and the second plot belongs to the negative samples. In positions 21-26, there is a high frequency of A and a low frequency of G in positive samples. For negative samples, it is the opposite.

**Fig 6:**
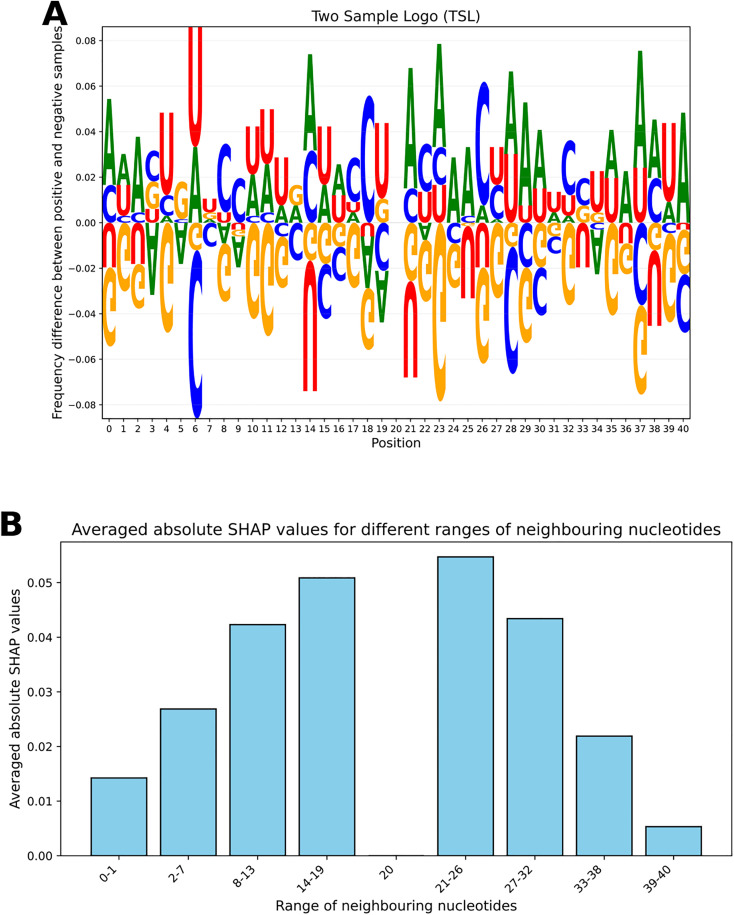
Visualization of positional nucleotide distributions and feature importance. A: Two Sample Logo (TSL) plot illustrating the nucleotide frequency differences between positive and negative samples. In positions 21-26, there is a high frequency of A and a low frequency of G in positive samples. For negative samples, it is the opposite. B: A bar plot displaying absolute SHAP values averaged over different ranges of nucleotide positions. The x-axis shows various ranges of neighboring nucleotides, while the y-axis shows the corresponding averaged absolute SHAP values.

Neighbourhood analysis on the samples was done in Figs 4A, 4B, 5, 6A and 6B. In Fig 4A, the nucleotide counts in upstream and downstream regions for both positive and negative samples are shown. The nucleotide counts were averaged across all RNA sequences. The upstream region was from position 0 to 19, and the downstream region was from position 21 to 40. From the plot, it is evident that the nucleotide counts in the upstream and downstream regions are almost the same. It applies to both positive and negative samples. However, nucleotide A counts in the upstream and downstream of the positive samples are greater than those of the negative samples, while the nucleotide G counts in these regions are lower for positive samples. It is more evident in Fig 4B where the averaged upstream and downstream nucleotide counts were summed, representing the nucleotide counts in the full region. The C and U counts are almost identical in both positive and negative samples. But the A count is greater, and the G count is less in positive samples than in the negative samples. In Fig 5, the nucleotide frequencies at each position across sequences for both positive and negative samples were illustrated. In nucleotide positions from 21 to 26, the frequency of nucleotide A is greater in positive samples than in negative samples. For the nucleotide frequency of G, it is the opposite. A Two Sample Logo (TSL) plot was depicted in Fig 6A. The TSL plot illustrates the nucleotide frequency differences between positive and negative samples. Even in the TSL plot, we can see that the frequency of nucleotide A is greater and the frequency of nucleotide G is lower in positive samples than in negative samples for nucleotide positions from 21 to 26. Therefore, nucleotide positions from 21 to 26 can be characterized as an A-rich and G-poor region for the positive samples and an A-poor and G-rich region for the negative samples.

In Fig 6B, a bar plot was depicted that displays absolute SHAP values averaged over different ranges of nucleotide positions. Firstly, the SHAP values were generated from the Inception branch of the proposed model, as this branch was responsible for extracting the local information from the RNA sequences. Then, the absolute SHAP values were averaged over different ranges of nucleotide positions. The values can be seen in Fig 6B. From the figure, it can be said that nucleotides in the positions from 21 to 26 highly contributed to the prediction of the InTrans-RNA5hmC model. To further test this, an XGB model was trained using the counts of A and G nucleotides from positions 21–26 as features. The model was trained on the Training set and tested on the Independent test set. It obtained 77.6% SN, 61.2% BACC, 66.7% F1 and 23.7% MCC. Although it gave inferior performance than the InTrans-RNA5hmC model, this result can provide further insight into the RNA 5hmC modification.

Several hypotheses can be drawn based on the obtained results: A-rich regions at positions 21–26 may show a structurally or functionally important segment of the RNA in the context of the 5hmC modification, since Adenine (A) is usually implicated in RNA binding and recognized by a variety of proteins. The identified A-rich motif at positions 21–26 is consistent with TET enzyme preferences for structurally accessible regions, as TET-mediated 5hmC formation exhibits context-dependent activity influenced by surrounding nucleotide composition [9,43]. A-rich sequences are commonly recognized by RNA-binding proteins such as hnRNPA1 and ELAVL family proteins, which regulate RNA stability and could potentially coordinate with 5hmC modification machinery [62,63]. The observed G-depletion at these positions may reflect avoidance of G-quadruplex-forming sequences, as these thermostable secondary structures can limit enzymatic accessibility and hinder modification processes [64,65].

Thus, in RNA 5hmC modification, the observed A/G ratio likely reflects a sequence or structural preference for recognition by the modification machinery. Enzymatic formation of RNA 5hmC by TET enzymes has indeed been demonstrated to have context-dependent activity, suggesting that modification efficiency can be affected by the surrounding nucleotide composition [9]. Moreover, transcriptome-wide mapping studies also showed that 5hmC is often present within specific sequence environments compatible with gene regulatory functions [11,43].

The enrichment of adenine at positions 21–26 in the positive samples may further suggest the involvement of RNA-processing enzymes or regulatory factors that bind preferentially to A-rich motifs. A-rich elements often feature in post-transcriptional regulatory mechanisms, such as splicing, stability, and subcellular localization, which may promote or stabilize the presence of 5hmC [66,67]. Taken together, these observations are in favor of the hypothesis that A-rich motifs represent preferred structural or recruitment sites that favor RNA 5hmC modification, while G-rich segments create structural constraints hindering modification.

## 4 Discussion

Different deep learning and ML models were compared and evaluated on the Validation set to choose the top-performing model as the proposed model. Both Validation and Independent test sets were used to thoroughly validate the proposed approach to prevent overfitting and guarantee that the model is generalizable. Maximum L2 norm constraints were applied, batch normalization was used to stabilize training, and dropout was incorporated to prevent the model from overfitting by randomly disabling units during training. These measures ensured that the model remained robust and did not overfit despite its complexity. Since the datasets have already been experimentally validated, further experimental validation is not required. The findings of the ablation study offer insights into the roles performed by each element of the proposed model.

The hypothesis that combining the Inception and Transformer architectures improves model accuracy by utilizing both local feature extraction and global contextual dependencies is supported by the InTrans-RNA5hmC model’s superior performance in the ablation study.

The neighborhood analysis provided some key insights. Nucleotide positions 21–26 can be classified as an A-rich and G-poor region for the positive samples and an A-poor and G-rich region for the negative samples based on the results of the neighborhood analysis. It is important to carry out more future investigations to determine whether the A/G ratio in positions 21–26 is a predictive marker of the presence of the RNA 5hmC modification or if it is simply related to specific RNA structural properties.

Additional research on the biological pathways or cellular processes involving RNA regions with low guanine content and high adenine content may help clarify how the 5hmC modification affects different regulatory networks or gene expression. By capturing local sequence patterns and global dependencies within RNA molecules, the learned features of the proposed model map to significant RNA properties. The Transformer branch tracks the long-range sequence interactions that facilitate RNA secondary structures. The Inception branch finds functional motifs associated with RNA processing, like splice sites and RNA-binding protein recognition. The contextual

information about nucleotide interactions offered by the Word embeddings and RiNALMo embeddings reveals the necessity of A-rich regions for 5hmC modification. By combining these properties, the model output links to significant biological mechanisms such as RNA splicing and post-transcriptional regulation.

The proposed model uses the resources as efficiently as possible despite having a complex deep-learning architecture. Although the model includes a stack of six Transformer encoder layers, the computational cost was minimized by employing a single Transformer-based branch in the architecture. Additionally, batch normalization and dropout layers were used to optimize the Inception branch. It guaranteed stability and computational efficiency throughout training. In terms of resource usage, the model’s parameters need about 370.62 MB of RAM. On a system running Pop! OS

22.04 LTS with an 11th Gen Intel Core i7 processor and 12 GB of RAM, the average inference time is 0.024 seconds. The model can be effectively implemented in real-world applications with negligible computational overhead. One potential concern is the limited size of the dataset. Due to the scarcity of publicly available RNA 5hmC datasets for *Drosophila melanogaster*, this study relied on a single specific dataset. However, based on the performance metrics on the independent test set, the proposed model shows promise for application to other RNA 5hmC datasets as they become available. Notably, recent state-of-the-art methods (Deep5hmC and XGB5hmC) also relied solely on this dataset. Future work can focus on further validating the model’s generalizability by applying it to additional datasets. Although RiNALMo is a 650M-parameter model, we do not retrain or fine-tune RiNALMo in this study; instead, we use its pre-trained embeddings as fixed feature representations, which substantially reduces the risk of overfitting. Another potential concern is that the pooled output of the Transformer stack, which uses six encoder layers, produces high-dimensional vectors (e.g., 41 × 639). High-dimensional representations can increase the risk of overfitting, particularly as the dataset is small in size. To mitigate overfitting from the high-dimensional Transformer outputs, we applied hyperparameter tuning (including epochs and dropout rates), used dropout layers throughout the network, and evaluated performance via 10-fold cross-validation (repeated 20 times with different random seeds) to ensure robust and generalizable results.

Averaging RiNALMo features for traditional ML models in Table 7 may have limited baseline performance due to information loss from dimensionality reduction. Recent studies [68,69] show that token- or residue-level embeddings, especially with attention-based models, better capture local sequence patterns. Although mean-pooling was adopted for efficiency and interpretability, alternative aggregation strategies (e.g., max-pooling or attention-weighted averaging) could lead to different comparative outcomes; future work should systematically assess the impact of feature aggregation choices on model performance.

## 5 Conclusions

In this study, a novel dual-branch deep learning model was designed. The two branches of InTrans-RNA5hmC were the Inception branch and the Transformer branch. Several feature descriptors were explored. After future selection, two feature descriptors were finalized. One was Word embeddings and the other was RiNALMo embeddings. Word embeddings were given as input to the Inception branch and RiNALMo embeddings were given as input to the Transformer branch. The feature sizes of Word embeddings and RiNALMo embeddings were (batch size, 41, 32) and (batch size, 41, 1280) for each sample, respectively. t-SNE and UMAP visualizations were utilized to demonstrate the model’s ability to learn meaningful and discriminative representations in the feature space. InTrans-RNA5hmC outperformed existing SOTA methods on the Independent test set in almost all performance metrics. It achieved 0.97 SN, 0.985 BACC, 0.985 F1, 0.985 AUC, and 0.993 AUPR on the Independent test set. A comprehensive sequence neighborhood analysis was conducted that revealed positions 21-26 to be A-rich in positive and G-rich in negative samples. The SHAP-based interpretability study confirmed that nucleotides in positions 21-26 are crucial in model predictions. Based on these observations, we hypothesize that positions 21-26 may represent a functionally relevant sequence motif for RNA 5hmC modification. However, this finding requires further experimental validation. To further strengthen the hypothesis of the importance of positions 21-26, an XGB model was trained using only A/G counts in this region as features, and its performance validates the hypothesis. Given that 5hmC-modified sites are A-rich, we can speculate that this region might be essential for transcription factors or RNA-binding proteins.

Future studies could leverage structural features derived from predicted RNA structures generated by advanced and complex RNA structure prediction models. It might improve the predictive performance of the model. Future researchers should examine the potential of the A/G ratio in positions 21–26 as a reliable predictive marker for RNA 5hmC modification. Further investigations are needed to determine if these patterns are related to RNA structural characteristics rather than the modification itself. Also, future research on the cellular functions and biological pathways involving areas with high adenine and low guanine content may help clarify the regulatory function of 5hmC. Future studies could enhance this research study by incorporating additional external datasets.

Potential RNA 5-Hydroxymethylcytosine modifications can be found using the predictions of the proposed model. For novel RNA sequences, these predicted regions can aid in guiding the search for RNA 5hmC modifications. New RNA 5hmC modifications and their functional implications in biological systems may be discovered more quickly as a result. It is an honest hope that the proposed model,

InTrans-RNA5hmC, will make significant contributions to the field of research on the prediction of RNA 5hmC modifications, given its importance in gene expression and complex regulatory networks, as well as its relationship to several human diseases like cancer, diabetes, and cardiovascular disorders.

## References

[1] Brosius J, Raabe CA. What is an RNA? A top layer for RNA classification. RNA Biol. 2016;13(2):140–4. doi: 10.1080/15476286.2015.1128064 26818079 PMC4829331

[2] Thiel V, Herold J, Schelle B, Siddell SG. Infectious RNA transcribed in vitro from a cDNA copy of the human coronavirus genome cloned in vaccinia virus. J Gen Virol. 2001;82(Pt 6):1273–81. doi: 10.1099/0022-1317-82-6-1273 11369870

[3] Williams GD, Gokhale NS, Horner SM. Regulation of Viral Infection by the RNA Modification N6-Methyladenosine. Annu Rev Virol. 2019;6(1):235–53. doi: 10.1146/annurev-virology-092818-015559 31283446 PMC6884077

[4] Uemura Y, Hasegawa A, Kobayashi S, Yokomori T. Tree adjoining grammars for RNA structure prediction. Theoretical Computer Science. 1999;210(2):277–303. doi: 10.1016/s0304-3975(98)00090-5

[5] Chen W, Feng P, Song X, Lv H, Lin H. iRNA-m7G: Identifying N7-methylguanosine Sites by Fusing Multiple Features. Mol Ther Nucleic Acids. 2019;18:269–74. doi: 10.1016/j.omtn.2019.08.022 31581051 PMC6796804

[6] Zhou Y, Zeng P, Li Y-H, Zhang Z, Cui Q. SRAMP: prediction of mammalian N6-methyladenosine (m6A) sites based on sequence-derived features. Nucleic Acids Res. 2016;44(10):e91. doi: 10.1093/nar/gkw104 26896799 PMC4889921

[7] Conde J, Yoon J-H, Roy Choudhury J, Prakash L, Prakash S. Genetic Control of Replication through N1-methyladenine in Human Cells. J Biol Chem. 2015;290(50):29794–800. doi: 10.1074/jbc.M115.693010 26491020 PMC4705973

[8] Liu Z-Y, Xing J-F, Chen W, Luan M-W, Xie R, Huang J, et al. MDR: an integrative DNA N6-methyladenine and N4-methylcytosine modification database for Rosaceae. Hortic Res. 2019;6:78. doi: 10.1038/s41438-019-0160-4 31240103 PMC6572862

[9] Fu L, Guerrero CR, Zhong N, Amato NJ, Liu Y, Liu S, et al. Tet-mediated formation of 5-hydroxymethylcytosine in RNA. J Am Chem Soc. 2014;136(33):11582–5. doi: 10.1021/ja505305z 25073028 PMC4140497

[10] Huber SM, van Delft P, Mendil L, Bachman M, Smollett K, Werner F, et al. Formation and abundance of 5-hydroxymethylcytosine in RNA. Chembiochem. 2015;16(5):752–5. doi: 10.1002/cbic.201500013 25676849 PMC4471624

[11] Roundtree IA, Evans ME, Pan T, He C. Dynamic RNA Modifications in Gene Expression Regulation. Cell. 2017;169(7):1187–200. doi: 10.1016/j.cell.2017.05.045 28622506 PMC5657247

[12] Haffner MC, Pellakuru LG, Ghosh S, Lotan TL, Nelson WG, De Marzo AM, et al. Tight correlation of 5-hydroxymethylcytosine and Polycomb marks in health and disease. Cell Cycle. 2013;12(12):1835–41. doi: 10.4161/cc.25010 23676216 PMC3735697

[13] Uribe-Lewis S, Carroll T, Menon S, Nicholson A, Manasterski PJ, Winton DJ, et al. 5-hydroxymethylcytosine and gene activity in mouse intestinal differentiation. Sci Rep. 2020;10(1):546. doi: 10.1038/s41598-019-57214-z 31953501 PMC6969059

[14] Douthwaite S, Kirpekar F. Identifying modifications in RNA by MALDI mass spectrometry. Methods Enzymol. 2007;425:3–20. doi: 10.1016/S0076-6879(07)25001-3 17673077

[15] Nees G, Kaufmann A, Bauer S. Detection of RNA modifications by HPLC analysis and competitive ELISA. Innate DNA and RNA Recognition: Methods and Protocols. 2014. p. 3–14. 10.1007/978-1-4939-0882-0_1 PMC7121164 24957224

[16] Grosjean H, Keith G, Droogmans L. Detection and quantification of modified nucleotides in RNA using thin-layer chromatography. RNA interference, editing, and modification: methods and protocols. 2004. p. 357–91. 10.1385/1-59259-775-0:357 15103084

[17] Kubista M, Andrade JM, Bengtsson M, Forootan A, Jonák J, Lind K. The real-time polymerase chain reaction. Molecular aspects of medicine. 2006;27(2–3):95–125. 16460794 10.1016/j.mam.2005.12.007

[18] Dong Z-W, Shao P, Diao L-T, Zhou H, Yu C-H, Qu L-H. RTL-P: a sensitive approach for detecting sites of 2’-O-methylation in RNA molecules. Nucleic Acids Res. 2012;40(20):e157. doi: 10.1093/nar/gks698 22833606 PMC3488209

[19] Liu Y, Chen D, Su R, Chen W, Wei L. iRNA5hmC: The First Predictor to Identify RNA 5-Hydroxymethylcytosine Modifications Using Machine Learning. Front Bioeng Biotechnol. 2020;8:227. doi: 10.3389/fbioe.2020.00227 32296686 PMC7137033

[20] Pisner DA, Schnyer DM. Support vector machine. Machine learning. Elsevier. 2020. p. 101–21.

[21] Ahmed S, Hossain Z, Uddin M, Taherzadeh G, Sharma A, Shatabda S, et al. Accurate prediction of RNA 5-hydroxymethylcytosine modification by utilizing novel position-specific gapped k-mer descriptors. Comput Struct Biotechnol J. 2020;18:3528–38. doi: 10.1016/j.csbj.2020.10.032 33304452 PMC7701324

[22] Nick TG, Campbell KM. Logistic regression. Topics in biostatistics. 2007. p. 273–301. 10.1007/978-1-59745-530-5_14 18450055

[23] Ali SD, Kim JH, Tayara H, Chong K to. Prediction of RNA 5-hydroxymethylcytosine modifications using deep learning. IEEE Access. 2021;9:8491–6. doi: 10.1109/access.2021.3049146

[24] Zhang S, Shi H. iR5hmcSC: Identifying RNA 5-hydroxymethylcytosine with multiple features based on stacking learning. Comput Biol Chem. 2021;95:107583. doi: 10.1016/j.compbiolchem.2021.107583 34562726

[25] Wibowo AS, Tayara H, Chong KT. XGB5hmC: Identifier based on XGB model for RNA 5-hydroxymethylcytosine detection. Chemometrics and Intelligent Laboratory Systems. 2023;238:104847. doi: 10.1016/j.chemolab.2023.104847

[26] Chen T, Guestrin C. Xgboost: A scalable tree boosting system. In: Proceedings of the 22nd ACM SIGKDD International Conference on Knowledge Discovery and Data Mining. 2016. 785–94.

[27] Khan S, Uddin I, Khan M, Iqbal N, Alshanbari HM, Ahmad B, et al. Sequence based model using deep neural network and hybrid features for identification of 5-hydroxymethylcytosine modification. Sci Rep. 2024;14(1):9116. doi: 10.1038/s41598-024-59777-y 38643305 PMC11551160

[28] Uddin I, Awan HH, Khalid M, Khan S, Akbar S, Sarker MR, et al. A hybrid residue based sequential encoding mechanism with XGBoost improved ensemble model for identifying 5-hydroxymethylcytosine modifications. Sci Rep. 2024;14(1):20819. doi: 10.1038/s41598-024-71568-z 39242695 PMC11379919

[29] Bepler T, Berger B. Learning the protein language: Evolution, structure, and function. Cell Syst. 2021;12(6):654-669.e3. doi: 10.1016/j.cels.2021.05.017 34139171 PMC8238390

[30] Elnaggar A, Heinzinger M, Dallago C, Rehawi G, Wang Y, Jones L, et al. ProtTrans: Toward Understanding the Language of Life Through Self-Supervised Learning. IEEE Trans Pattern Anal Mach Intell. 2022;44(10):7112–27. doi: 10.1109/TPAMI.2021.3095381 34232869

[31] Penić RJ, Vlašić T, Huber RG, Wan Y, Šikić M. RiNALMo: general-purpose RNA language models can generalize well on structure prediction tasks. Nat Commun. 2025;16(1):5671. doi: 10.1038/s41467-025-60872-5 40593636 PMC12219582

[32] Chen J, Hu Z, Sun S, Tan Q, Wang Y, Yu Q. Interpretable RNA foundation model from unannotated data for highly accurate RNA structure and function predictions. arXiv preprint. 2022. doi: 10.48550/arXiv.2204.00300

[33] Wang X, Gu R, Chen Z, Li Y, Ji X, Ke G. UNI-RNA: universal pre-trained models revolutionize RNA research. bioRxiv. 2023.

[34] Pokharel S, Pratyush P, Heinzinger M, Newman RH, Kc DB. Improving protein succinylation sites prediction using embeddings from protein language model. Sci Rep. 2022;12(1):16933. doi: 10.1038/s41598-022-21366-2 36209286 PMC9547369

[35] Soylu NN, Sefer E. DeepPTM: Protein Post-translational Modification Prediction from Protein Sequences by Combining Deep Protein Language Model with Vision Transformers. CBIO. 2024;19(9):810–24. doi: 10.2174/0115748936283134240109054157

[36] Szegedy C, Liu W, Jia Y, Sermanet P, Reed S, Anguelov D, et al. Going Deeper with Convolutions. In: Proceedings of the IEEE Conference on Computer Vision and Pattern Recognition. 2015. 1–9.

[37] Vaswani A, Shazeer N, Parmar N, Uszkoreit J, Jones L lion, Gomez AN, et al. Attention is all you need. Advances in neural information processing systems. 2017;30.

[38] Soylu NN, Sefer E. BERT2OME: Prediction of 2’-O-Methylation Modifications From RNA Sequence by Transformer Architecture Based on BERT. IEEE/ACM Trans Comput Biol Bioinform. 2023;20(3):2177–89. doi: 10.1109/TCBB.2023.3237769 37819796

[39] Van der Maaten L, Hinton G. Visualizing data using t-SNE. Journal of Machine Learning Research. 2008;9(11).

[40] McInnes L, Healy J, Melville J. Umap: Uniform manifold approximation and projection for dimension reduction. arXiv preprint. 2018. doi: 10.48550/arXiv.1802.03426

[41] Scott M, Su-In L. A unified approach to interpreting model predictions. Advances in Neural Information Processing Systems. 2017;30:4765–74.

[42] Vacic V, Iakoucheva LM, Radivojac P. Two Sample Logo: a graphical representation of the differences between two sets of sequence alignments. Bioinformatics. 2006;22(12):1536–7. doi: 10.1093/bioinformatics/btl151 16632492

[43] Delatte B, Wang F, Ngoc LV, Collignon E, Bonvin E, Deplus R, et al. RNA biochemistry. Transcriptome-wide distribution and function of RNA hydroxymethylcytosine. Science. 2016;351(6270):282–5. doi: 10.1126/science.aac5253 26816380

[44] Huang Y, Niu B, Gao Y, Fu L, Li W. CD-HIT Suite: a web server for clustering and comparing biological sequences. Bioinformatics. 2010;26(5):680–2. doi: 10.1093/bioinformatics/btq003 20053844 PMC2828112

[45] Dao T. Flashattention-2: Faster attention with better parallelism and work partitioning. arXiv preprint. 2023. doi: 10.48550/arXiv.230708691

[46] Shazeer N. Glu variants improve transformer. arXiv preprint. 2020. doi: arXiv:200205202

[47] Su J, Ahmed M, Lu Y, Pan S, Bo W, Liu Y. RoFormer: Enhanced transformer with Rotary Position Embedding. Neurocomputing. 2024;568:127063. doi: 10.1016/j.neucom.2023.127063

[48] Chen Z, Zhao P, Li F, Marquez-Lago TT, Leier A, Revote J, et al. iLearn: an integrated platform and meta-learner for feature engineering, machine-learning analysis and modeling of DNA, RNA and protein sequence data. Brief Bioinform. 2020;21(3):1047–57. doi: 10.1093/bib/bbz041 31067315

[49] Altman DG, Bland JM. Diagnostic tests. 1: Sensitivity and specificity. BMJ. 1994;308(6943):1552. doi: 10.1136/bmj.308.6943.1552 8019315 PMC2540489

[50] Powers DM. Evaluation: from precision, recall and F-measure to ROC, informedness, markedness and correlation. arXiv preprint. 2020. doi: 10.48550/arXiv.2010.16061

[51] Fawcett T. An introduction to ROC analysis. Pattern Recognition Letters. 2006;27(8):861–74. doi: 10.1016/j.patrec.2005.10.010

[52] Keilwagen J, Grosse I, Grau J. Area under precision-recall curves for weighted and unweighted data. PLoS One. 2014;9(3):e92209. doi: 10.1371/journal.pone.0092209 24651729 PMC3961324

[53] Yu M, Yuan Z, Li R, Shi B, Wan D, Dong X. Interpretable machine learning model to predict surgical difficulty in laparoscopic resection for rectal cancer. Front Oncol. 2024;14:1337219. doi: 10.3389/fonc.2024.1337219 38380369 PMC10878416

[54] Ghosh SK, Khandoker AH. Investigation on explainable machine learning models to predict chronic kidney diseases. Sci Rep. 2024;14(1):3687. doi: 10.1038/s41598-024-54375-4 38355876 PMC10866953

[55] Wei C, Wang J, Yu P, Li A, Xiong Z, Yuan Z, et al. Comparison of different machine learning classification models for predicting deep vein thrombosis in lower extremity fractures. Sci Rep. 2024;14(1):6901. doi: 10.1038/s41598-024-57711-w 38519523 PMC10960026

[56] Joe H, Kim H-G. Multi-label classification with XGBoost for metabolic pathway prediction. BMC Bioinformatics. 2024;25(1):52. doi: 10.1186/s12859-024-05666-0 38297220 PMC10832249

[57] Lin L i, Long Y ao, Liu J inkai, Deng D ongliang, Yuan Y u, Liu L ubin, et al. FRP-XGBoost: Identification of ferroptosis-related proteins based on multi-view features. International Journal of Biological Macromolecules. 2024;262:130180. 38360239 10.1016/j.ijbiomac.2024.130180

[58] Rumelhart DE, Hinton GE, Williams RJ. Learning representations by back-propagating errors. Nature. 1986;323(6088):533–6. doi: 10.1038/323533a0

[59] Hochreiter S, Schmidhuber J. Long short-term memory. Neural Comput. 1997;9(8):1735–80. 9377276 10.1162/neco.1997.9.8.1735

[60] Cortes C. Support-vector networks. Machine learning. 1995.

[61] Peterson L. K-nearest neighbor. Scholarpedia. 2009;4(2):1883. doi: 10.4249/scholarpedia.1883

[62] Dominguez D, Freese P, Alexis MS, Su A, Hochman M, Palden T, et al. Sequence, structure, and context preferences of human RNA binding proteins. Mol Cell. 2018;70(5):854-867.e9. doi: 10.1016/j.molcel.2018.05.001 29883606 PMC6062212

[63] Ray D, Kazan H, Cook KB, Weirauch MT, Najafabadi HS, Li X, et al. A compendium of RNA-binding motifs for decoding gene regulation. Nature. 2013;499(7457):172–7. doi: 10.1038/nature12311 23846655 PMC3929597

[64] Kwok CK, Merrick CJ. G-Quadruplexes: Prediction, Characterization, and Biological Application. Trends Biotechnol. 2017;35(10):997–1013. doi: 10.1016/j.tibtech.2017.06.012 28755976

[65] Millevoi S, Moine H, Vagner S. G-quadruplexes in RNA biology. Wiley Interdiscip Rev RNA. 2012;3(4):495–507. doi: 10.1002/wrna.1113 22488917

[66] Matoulkova E, Michalova E, Vojtesek B, Hrstka R. The role of the 3’untranslated region in post-transcriptional regulation of protein expression in mammalian cells. RNA biology. 2012;9(5):563–76. 22614827 10.4161/rna.20231

[67] Tian B, Manley JL. Alternative polyadenylation of mRNA precursors. Nat Rev Mol Cell Biol. 2017;18(1):18–30. doi: 10.1038/nrm.2016.116 27677860 PMC5483950

[68] Li L, Li X, Wu H. A novel deep learning framework with dynamic tokenization for identifying chromatin interactions along with motif importance investigation. Briefings in Bioinformatics. 2025;26(3). 10.1093/bib/bbaf289 PMC12204613 40536817

[69] Zhou X, Wu H. scHiClassifier: a deep learning framework for cell type prediction by fusing multiple feature sets from single-cell Hi-C data. Briefings in Bioinformatics. 2024;26(1). 10.1093/bib/bbaf009 PMC11744636 39831891

